# New Appliances & Things Medical

**Published:** 1910-07-23

**Authors:** 


### NEW APPLIANCES & THINGS MEDICAL.

**[We shall be glad to receive at our Office, 28 & 29 Southampton Street, Strand, London, W.C., from the manufacturers, specimens of all new preparations and appliances.]**

### CALSA.

(Torbet, Ltd., 10 Bush Lane, London, E.C.)

Calsa is the proprietary name of a new agar-agar preparation, purified and sterilised by a special process. In appearance Calsa reminds us of the finest samples of pure Indian Ocean agar, as it is practically tasteless and odourless. It is probably the purest form of this seaweed on the market. No laxative has been added to it, and it has been felt that if such is necessary, its choice and dose should be left to the discretion of the prescriber. It is put up in a neat cardboard box, the 2s. size being sufficient for six adult doses. The preparation is an excellent form of taking agar and can be confidently recommended. Each compartment in the box contains the daily dose for an adult, although at first considerably less is usually taken. Special attention is directed to the recipe booklet accompanying each package, in which are to be found various means for preparing the Calsa in palatable form.

### SPIROSAL.

(The Bayer Co., Limited, 19 St. Dunstan's Hill, London, E.C.)

Spirosal is an ester of salicylic acid, intended for external application. It is a colourless, practically odourless liquid, readily soluble in alcohol or ether and slightly soluble in water, and is most efficiently diluted with rectified spirit of wine when used for external application. It has been found useful in cases of lumbago, arthritis, and allied conditions, and is remarkably well borne by ordinary skins. When Spirosal is rubbed in, salicylic acid is rapidly absorbed and the therapeutic effect so obtained is considerable. The preparation is put up in ounce bottles and is well worth a trial.

